# Interfering RNA and HIV: Reciprocal Interferences

**DOI:** 10.1371/journal.ppat.1000162

**Published:** 2008-09-26

**Authors:** Pierre Corbeau

**Affiliations:** Laboratoire d'Immunologie, Hôpital Carémeau, Nîmes and Institut de Génétique Humaine, CNRS UPR1142, Montpellier, France; University of British Columbia, Canada

## Abstract

In this review, a quick presentation of what interfering RNA (iRNA) are—small RNA able to exert an inhibition on gene expression at a posttranscriptional level, based on sequence homology between the iRNA and the mRNA—will be given. The many faces of the interrelations between iRNA and viruses, particularly HIV, will be reviewed. Four kinds of interactions have been described: i) iRNA of viral origin blocking viral RNA, ii) iRNA of viral origin downregulating cellular mRNA, iii) iRNA of cellular origin (microRNA) targeting viral RNA, and iv) microRNA downregulating cellular mRNA encoding cell proteins used by the virus for its replication. Next, HIV strategies to manipulate these interrelations will be considered: suppression of iRNA biosynthesis by Tat, trapping by the HIV TAR sequence of a cell component, TRBP, necessary for iRNA production and action, and induction by the virus of some microRNA together with suppression of others. Then, we will discuss the putative effects of these mutual influences on viral replication as well as on viral latency, immune response, and viral cytopathogenicity. Finally, the potential consequences on the human infection of genetic polymorphisms in microRNA genes and the therapeutic potential of iRNA will be presented.

## Introduction

The discovery that cells produce small RNAs called interfering RNAs (iRNAs) that are able to inhibit in a sequence-specific way gene expression at a post-transcriptional level, a phenomenon called RNA interference, is a recent breakthrough in biology. This discovery has already had and will have many consequences on our knowledge of cell physiology and pathology, on our ability to regulate protein production at the laboratory, using tailored small iRNAs (siRNAs), and soon in medicine. Cohabitation of iRNAs physiologically produced by human cells, called microRNAs (miRNAs), and viral RNAs in the same cell results in interactions that may have an impact on virus replication, host cell physiology, and anti-microbial immune response. In this article, we will describe briefly the miRNA machinery, review the various connections existing between iRNAs and HIV RNAs, evaluate their consequences for both actors, and consider how we could interfere with these connections in order to regulate virus infection.

## Human Cellular miRNAs

**Figure 1:**
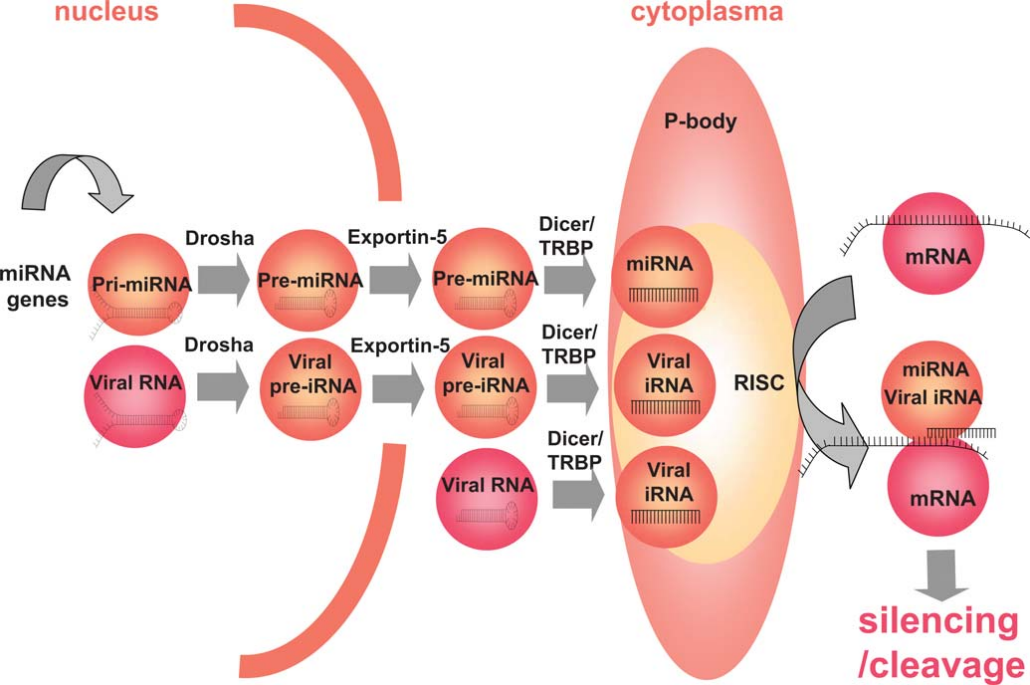
Biosynthesis and Activity of iRNAs

miRNAs are transcribed by intergene regions, introns, and even exons, either as polycistronic transcripts if they are clustered or as monocistronic transcripts if they are not [1]–[3]. These transcripts, called pri-miRNAs, are imperfect RNA hairpins of hundreds to thousands of base pairs [4]. They are processed in the nucleus by the Rnase III endonuclease Drosha to a stem loop structure of about 60 base pairs, the pre-miRNA (Figure 1) [5]. The transfer of pre-miRNA from the nucleus to the cytoplasma is mediated by the nuclear export factor exportin 5 [6]. There, pre-miRNAs are cleaved by another Rnase III endonuclease, Dicer [7]. Dicer delivers an approximately 22–base pair duplex. One strand of this duplex, the mature miRNA, still bound to Dicer, is driven towards ribosome-free compartments of the cytoplasma called P-bodies (processing bodies) by an association of molecules called RISC (RNA-induced silencing complex), which includes the endonuclease Argonaute-2 (Ago-2) [8]. The targets of the miRNA-loaded RISC are the RNAs presenting with sequence homology with nucleotides 2–7 in the 5′ portion of the miRNA. Most of the time, the consequence is an inhibition of translation of the mRNA, and sometimes, particularly if the match between the mRNA and the miRNA is perfect, the mRNA is cleaved. Of note, TAR RNA–binding protein (TRBP), a cell protein initially discovered for its capacity to bind to the TAR sequence of HIV RNA, also binds to Dicer and Ago-2, and is necessary for the maturation of pre-miRNA into miRNA as well as for interfering RNA function [9],[10].

A single miRNA targets at least 100 transcripts from various genes, and one mRNA may be targeted, at its 3′ end, by different miRNAs [11]. Thus, miRNAs, whose number has been estimated to be 340 [12], and more recently over 600 (http://microrna.sanger.ac.uk/cgi-bin/sequences/mirna_summary.pl?org=hsa), could regulate at least one-third of all human genes [13].

Researchers have taken advantage of this pathway to induce the specific destruction of mRNAs in order to silence genes of choice. They do so either by directly transfecting siRNAs of approximately 21 base pairs, or by delivering transgenes encoding hairpin RNAs, small hairpin RNAs (shRNAs) processed by Dicer to siRNAs, with a stretch perfectly complementary with the target mRNA. Now, it happens that some viral RNAs adopt a stem loop conformation recognized by Drosha or Dicer as a suitable substrate and are processed to iRNAs, and we will call these viral iRNAs (viRNAs) to distinguish them from the endogenous cellular miRNAs. As the characteristics of Drosha and Dicer substrates are not fully defined, the fact that a viral RNA will or will not be processed to an iRNA cannot at present be accurately predicted. Likewise, the characteristics that make an mRNA a target for a given iRNA, including its own structure [14],[15], its accessibility, i.e., its localization in the cell and its association with other components of the cell [16], are not absolutely established. Consequently, any bioinformatic study predicting that a viral RNA will be processed into viRNA or that an mRNA will be silenced by an iRNA has to be confirmed experimentally. It must also be kept in mind that many studies analyze the effect on viral RNA of iRNAs at high concentrations obtained after transfection, and that such results must be confirmed in the course of the infection at physiological concentrations of iRNAs.

## RNA Interferences in the Cell/Virus Complex

**Figure 2:**
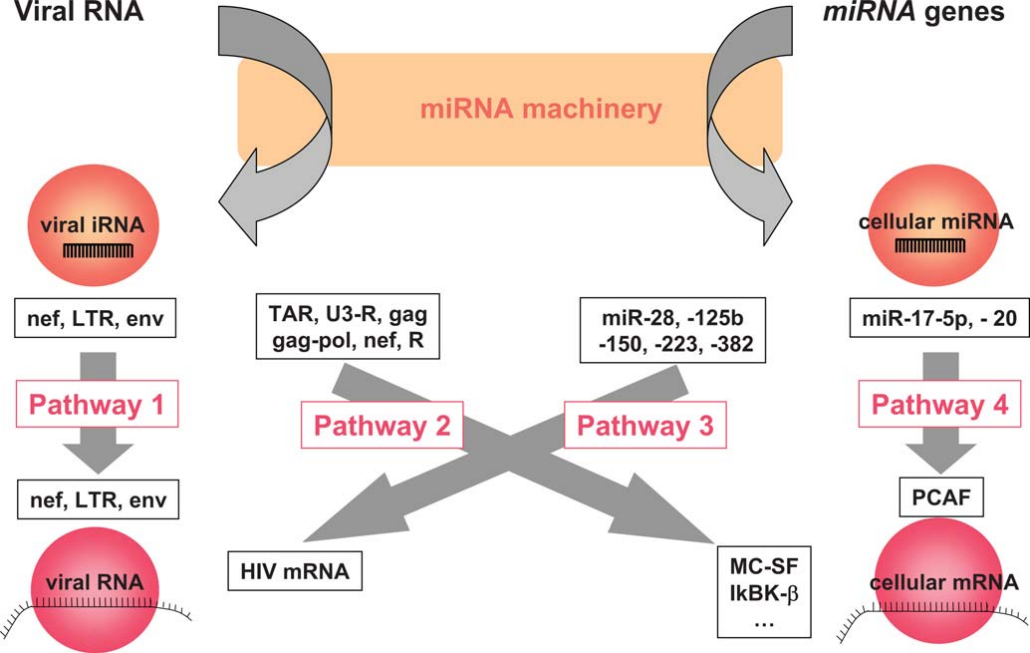
Interactions between iRNAs, Cellular mRNAs, and HIV RNAs

Viral RNAs and the miRNA machinery may interfere in various ways (Figure 2). First, the viRNAs generated by the cell from virus RNAs may target back viral RNAs (pathway 1), but also cell mRNAs that happen to share some sequence homology with them (pathway 2). Second, cellular miRNAs may recognize viral RNAs and silence them (pathway 3). Finally, the cell may produce miRNAs that control the expression of a cellular protein necessary for the virus life cycle (pathway 4). We will review these various possibilities, comparing examples of what is already known for other viruses with what is known for HIV.

### 

#### Autosilencing: viRNA against viral RNA (pathway 1)

Obviously, some viRNAs will match exactly with some viral RNAs issued from the same genomic sequence, and induce their destruction. This is the case, for instance, in the course of simian virus 40 (SV40) infection. The virus encodes two RNAs with a stem loop structure that are transformed by the host cell into miR-S1s that are complementary to mRNA coding for the viral T antigen. Consequently, T antigen mRNA is degraded [17]. In this scenario, host miRNA machinery turns viral RNA against viral RNA. The same phenomenon has been looked for in HIV infection. A precursor [18] and a recent [19] study by two major groups in the field failed to identify iRNA of HIV origin in infected cells. Yet, a nef- and LTR-specific HIV miRNA able to inhibit LTR-driven transcription has been evidenced by another group [20],[21]. Moreover, a fourth group has reported that a stem loop HIV RNA can be processed by Dicer to a viRNA able to target env mRNA and that transfection of the corresponding shRNA inhibited over 80% of env mRNA production [22], a result that was challenged later [19]. Finally, Klase et al. [23] and Ouellet et al. [24] have shown that TAR is a source of viRNAs: Dicer interacts with TAR, and cleaves it to produce TAR-derived viRNAs able to exert gene downregulatory effects. The level of anti-HIV activity of this pathway, which is controversial, remains to be evaluated in the context of the infection. Anyway, the antiviral effect of HIV viRNAs is at best partial.

#### viRNA against cell mRNA (pathway 2)

It may happen that the host cell transforms a viral RNA into a viRNA with some degree of homology with a cell mRNA. In this case, the viRNA will inhibit the expression of the corresponding cellular gene. Such a possibility has been recently described during human cytomegalovirus (HCMV) infection. HCMV encodes an RNA transformed by the infected cell into a viRNA, hcmv-miR-UL112, that blocks the translation of major histocompatibility complex class I–related chain B (MICB). Of note, MICB is a stress-induced molecule expressed on HCMV-infected cells that is recognized by the natural killer (NK) cell activating receptor NKG2D [25]. Likewise, are there viRNAs of HIV origin able to target cell mRNAs? Computer-directed analyses have evidenced regions of base complementarity between HIV-1 sequences and human genes involved in HIV infection, e.g., *CD4, CD28, CD40L, IL-2, IL-3, IL-12,* and *TNFβ* [26]. Moreover, Bennasser et al. have predicted the existence of five HIV RNAs that could be transformed by Dicer into five viRNAs able to target various cellular mRNAs [27]. If these predictions are experimentally confirmed, HIV could also manipulate cellular biology and host immune response via viRNAs that were produced by the infected cell.

#### Cellular miRNA against virus RNA (pathway 3)

Sometimes the cell does not borrow viral RNAs to produce viRNAs with antiviral activity, but rather uses its own miRNAs. A striking example of this strategy is represented by the cell miR-32 that targets the open reading frame 2 of the primate foamy virus type 1, thereby inhibiting virus translation [28]. In this way, a cellular miRNA may directly target a virus RNA and block virus production. Interestingly, the same process results in the opposite effect for hepatits C virus (HCV). The endogenous miR-122, which is specifically produced by liver cells, binds to the 5′ noncoding region of HCV, but, unusually, this results in an increase in viral RNA replication through a mechanism that remains to be unraveled [29]. Interactions between cellular miRNAs and HIV RNAs may also exist, in so far as an in silico study predicted that five human miRNAs, expressed in T cells, might target nef, vpr, vif, and env RNAs [30]. Recently, Huang et al. have shown that miR-28, -125b, -150, -223, and -382, cellular miRNAs that are overexpressed in quiescent T4 lymphocytes, target sequences in the 3′ end of HIV-1 RNA, silencing thus almost all viral messengers [31]. Neutralizing these cellular miRNAs by transfecting specific antagonists into nonactivated T4 cells from patients with HIV under highly active antiretroviral therapy increased by 10-fold the in vitro efficiency of virus isolation. These observations strongly argue for a role of cellular miRNAs in HIV latency. This hypothesis, together with the suppression exerted by HIV on miRNA biosynthesis, might partly explain why HIV has not mutated to escape from the inhibitory effect of iRNAs of viral or cellular origin.

#### Cellular miRNA targeting cell mRNA encoding proteins involved in virus replication (pathway 4)

**Figure 3:**
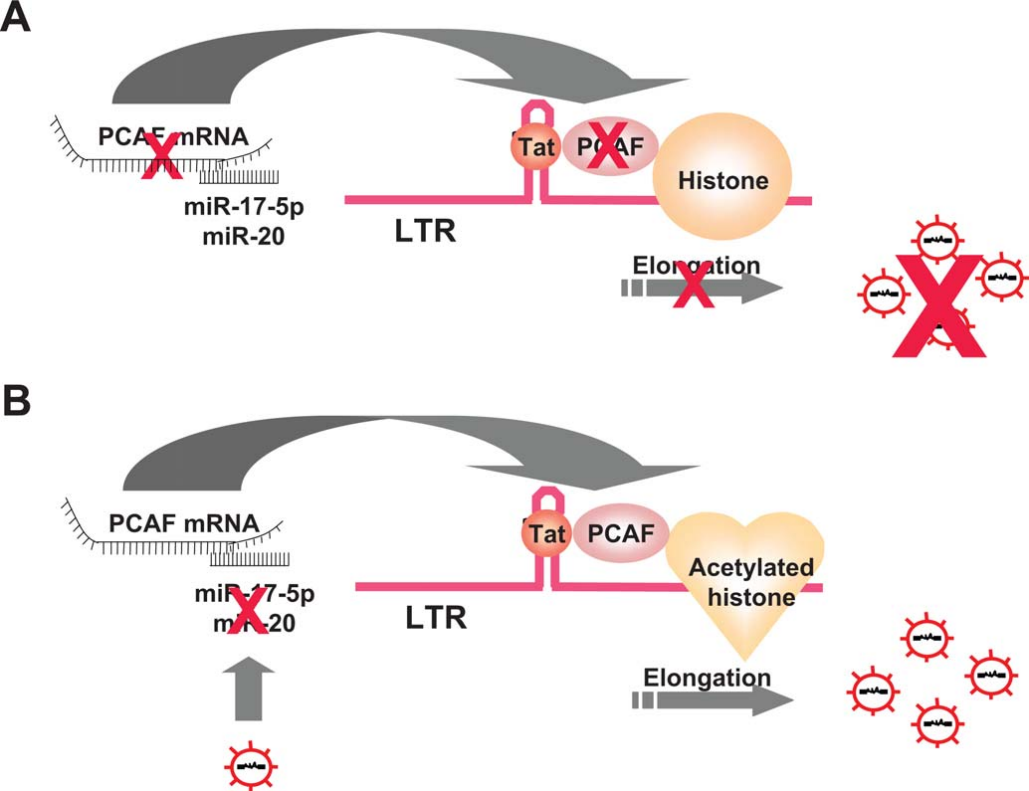
Inhibition of HIV Expression by the Silencing Exerted by miR-17-5p and miR-20 on PCAF (A), and Downregulation of This Inhibition by HIV (B)

Finally, a fourth situation, where host miRNAs limit viral proliferation by acting on cellular mRNA, has been recently proposed for HIV [32]. Triboulet et al. have shown that the cellular miRNAs miR-17-5p and miR-20 silence the mRNA encoding the histone acetylase PCAF. PCAF has been previously presented as a host cofactor for Tat transactivation of HIV LTR, and as being recruited by Tat and remodeling the histone architecture in the vicinity of the LTR, promoting thereby HIV gene expression (Figure 3A). This is to say that human cells seem to permanently downregulate HIV-1 replication by depriving the infected cell of an endogenous enzyme that could be necessary for virus gene expression.

## Regulation of the miRNA Pathway by the Virus

The few examples we have just reviewed suggest that, most of the time, the miRNA machinery works against the virus. It is not surprising then, that many viruses have elaborated strategies to subvert this machinery. And HIV is not the last one.

### 

#### Inhibition of miRNA production: viral suppressors of RNA silencing

**Figure 4:**
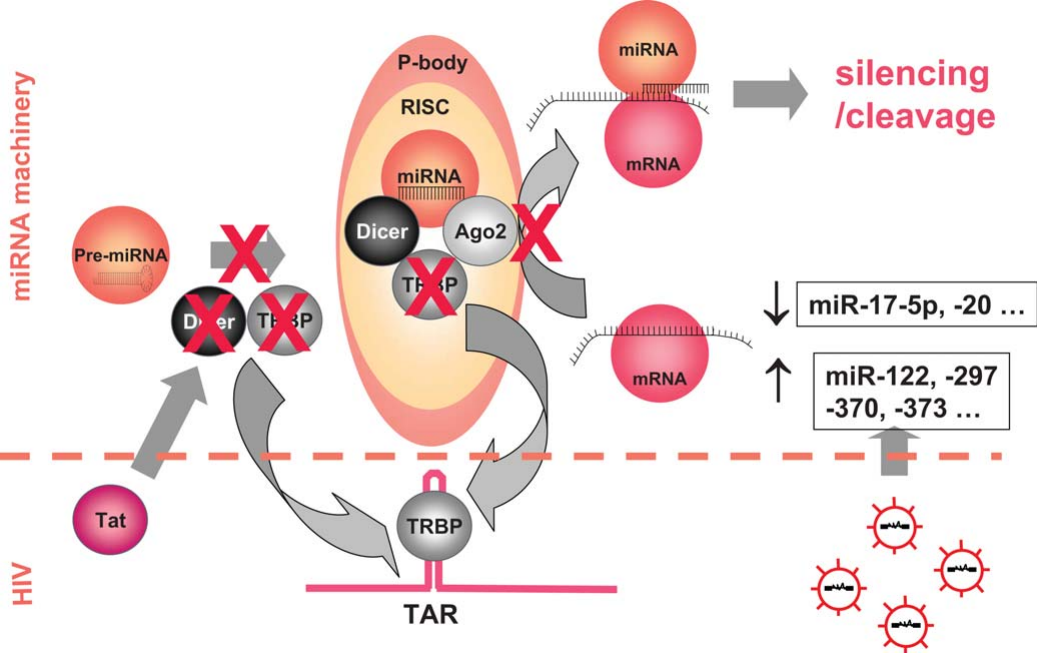
Regulation of the miRNA Machinery by HIV

A strategy adopted by many viruses involves binding to a component of the miRNA machinery in order to block it. For instance, the adenoviral protein VA1 inhibits the nucleo-cytoplasmic transport of pre-miRNA by forming a complex with exportin 5 [33]. HIV seems to target two other factors of RNA silencing, Dicer and TRBP (Figure 4). Bennasser et al. have shown that purified Tat protein inhibits the capacity of Dicer to process double-stranded RNA to short iRNAs in vitro [22]. Moreover, the TAR and RRE sequences of HIV RNA, by recruiting TRBP, could competitively inhibit the effect of TRBP on pre-miRNA processing and on miRNA function [34],[35]. Yet, the capacity of Tat to hinder miRNA biogenesis has been recently challenged [19], and the impact on HIV infection of these inhibitions of miRNA expression remains to be quantified. Anyhow, if HIV encodes suppressors of RNA silencing, their effect is obviously incomplete.

This double mechanism of suppression of RNA silencing strongly suggests that the interest of HIV is to counteract RNA interference; that is to say, the effect of the miRNA machinery is globally harmful for the virus. In support of this notion, a point mutation in Tat, which abrogates its inhibitory effect on Dicer, but not its transactivation effect on HIV LTR, results in a reduction in virus replication [22]. Likewise, Haasnoot et al. have shown that a Tat protein able to block Dicer activity is necessary for efficient HIV production [36]. Moreover, Triboulet et al. have reported that the inhibition of Drosha or Dicer using specific siRNA increases HIV replication in peripheral blood mononuclear cells from individuals with HIV [32].

#### Viral up- and downregulation of miRNA concentrations

In addition to globally diminishing miRNA production, viruses may also specifically regulate, positively or negatively, the level of expression of some miRNAs. This is the case for tobacco mosaic virus, where infection results in an increase in miR-156, -160, -164, -166, -169, and -171 in *Nicotiana tabacum* [37]. HIV infection also results in such a phenomenon. Triboulet et al. have reported that the infection of a lymphoid cell line by HIV-1 downregulates six miRNAs, including miR-17-5p and miR-20, and upregulates eleven others, including miR-122, -297, -370, and -373 [32]. The mechanism responsible for this selective regulation by HIV remains to be unveiled. During tobacco mosaic virus infection, it is the accumulation of two viral proteins, the movement protein and the coat protein, that is responsible for the change in miRNAs concentrations [37]. Of note, two of the miRNAs downregulated during HIV infection are precisely miR-17-5p and miR-20, which target the Tat cofactor PCAF. Thus, by reducing the amount of miR-17-5p and miR-20 available, the virus alleviates the negative control exerted by the cell on PCAF, thereby facilitating its own transcription (Figure 3B).

## Consequences of the Interactions between iRNAs and the Other RNAs during Infection

Thus, viruses and the miRNA machinery of the host cell interact in various ways. These interactions may have consequences on the replication and the pathogenicity of the virus, but also on the immune response of the host.

### 

#### Consequences for virus replication/latency

Interactions between iRNAs and other RNAs in the infected cell may have consequences on the virus life cycle, mostly negative, with one exception, HCV. For HIV, viRNA complementary to env, nef, and/or LTR sequences [20]–[22] and cellular miRNAs targeting the 3′ end of HIV RNAs [31] could inhibit virus replication. Moreover, the cellular miR-17-5p and miR-20, through their repression of PCAF expression (Figure 3B), seem to exert the same effect [32]. In these various ways, RNA interference might downregulate HIV production. Logically, the virus tries to counter this effect by blocking Dicer activity and by hijacking TRBP.

The global impact on in vivo infection of the interplay between virus and cell RNAs, and the relative importance of each interaction, remain to be determined. This interplay could influence the efficiency of HIV replication, and thereby the rate of disease progression, but it could also be involved in HIV latency and in the constitution of the viral reservoir. Finally, differences in the anti-HIV efficiency of RNA inteference could exist between individuals. These differences might be due to genetic polymorphisms in sequences regulating miRNA gene transcription or stability, as well as polymorphisms in cellular miRNA sequences resulting in variations in processing or targeting as already described for miR-138-2 and miR-30c-2 [38].

#### Consequences on the immune response

Inhibition of virus gene expression by RNA interference may downregulate virus replication in vitro, but in vivo, this downregulation might help the virus to escape from the immune response. This is the case for the above-mentioned inhibition exerted by the SV40 miRNAs miR-S1s on the T antigen (see “Autosilencing: viRNA against virus RNA (pathway 1)”). This inhibition occurs late enough in the virus life cycle not to hinder SV40 replication, but early enough to reduce T lymphocyte cytotoxicity and interferon-γ production triggered by the presence of the viral T antigen [17]. The targeting of cell mRNA by viRNA (pathway 2) may also result in the reduction of immune response. This possibility is illustrated by the inhibition of MICB, a ligand for NK cell activating receptor, by an HCMV miRNA (“viRNA against cell mRNA (pathway 2)”). This targeting is not innocent, since it results in a decrease in antiviral NK activity. Some cellular miRNAs are involved in innate immunity, e.g., miR-155 induced by Toll-like receptor ligands or interferon-β (IFN-β) [39] and miRNA let-7a mediating IL-6-induced cell survival [40]. Some cellular miRNAs are involved in specific immunity, e.g., miR-155 regulating genes driving functions of lymphocytes [41],[42], miR-150 controlling the differenciation of B cells via c-Myb [43], and miR-181a modulating the antigen sensitivity of T cells [44]. A striking example of the role played by miRNAs in immunity has been lately given by Pedersen et al. This group has reported that some of the anti-HCV effect of IFN-β is mediated by five cellular miRNAs, induced by IFN-β, that target HCV RNA [45]. Consequently, changes in miRNA expression induced by HIV might also alter the immune response.

#### Consequences on the pathogenicity

These virus/cell, iRNA/RNA interactions could also have consequences on the pathogenicity of the infection. Because cellular miRNAs regulate major mechanisms of cell physiology, such as proliferation, differentiation, apoptosis, or tumorogenicity [46], targeting of cell mRNAs by viRNAs (pathway 2) and/or specific or global virus-induced disturbance of the cellular miRNA production might have pathogenic effects. The extent to which the modifications of host biology observed in individuals with HIV are mediated by RNA interference remains to be addressed, however.

## Therapeutic Opportunities

Besides strategies using synthetic interfering RNAs (siRNAs or shRNAs) to target mRNA encoding cell proteins necessary for virus replication or directly target HIV RNA, the knowledge of HIV RNA/RNA silencing connections might pave the way for new therapeutic approaches. The fact that siRNAs or siRNAs antagonists modified chemically for stability and conjugated to cholesterol or liposomes have a specific, long-lasting, and ubiquitous effect when administrated intravenously [47],[48] is particularly encouraging for this kind of approach.

As interfering RNAs disturb HIV replication, the possibility to act on the viral infection through miRNAs must be considered. At least two opposite strategies may be proposed. First, the miRNA machinery could be manipulated in order to hinder virus replication. Second, because of the involvement of miRNA in HIV latency, the miRNA machinery could be manipulated in order to provoke virus replication in the reservoir cells with the aim of lysing them. This second approach has the advantage of only requiring a brief treatment that is sufficient to induce HIV expression in infected, non-productive quiescent cells. To reach this goal, a possibility is to target host miRNA. For instance, the neutralization of miR-17 and miR-20 by specific inhibitors should release the inhibition exerted by these miRNAs on PCAF and hopefully trigger HIV replication in reservoir cells, resulting in the eradication of this population. A drawback of such a strategy is that the inhibition of a given miRNA will increase the level of expression of all of its mRNA targets with possible side effects. As our knowledge on the impact of the disturbance induced by HIV on host immunity and cell physiology increases, new therapeutic strategies may arise.

Last, the manipulation of miRNAs in order to boost HIV production in vitro may ameliorate the efficiency of virus recovery from patients' cells [31],[32], and of virus mass production.

## Conclusion

The miRNA pathway appears to be a new branch of natural antiviral immunity. Understanding the complex relationship that exists between the miRNA pathway and HIV will enlighten the physiopathology of the infection and offer new therapeutic strategies. As miRNA might be involved in HIV replication and latency, pathogenicity, and immune response, manipulating miRNA might enable us to modulate all of these aspects of the infection. Much remains to be discovered with surprises on the way. For instance, the eventuality that miRNA might also regulate DNA transcription in mammal cells [49] by inducing DNA methylation [50] and/or other mechanisms [51],[52] could offer additional means to inhibit HIV replication, as suggested by recent data [23],[53].
